# Evaluating the effect of smoking and its cessation on semen parameters

**DOI:** 10.1530/RAF-24-0135

**Published:** 2025-10-29

**Authors:** Ahmed Ragheb, Ahmed Abdelbary, Amr Massoud, Mahmoud Abd Elkhalek, Ahmed Elbatanony

**Affiliations:** ^1^ Faculty of Medicine, Beni-Suef University, Urology, Beni-Suef, Egypt; ^2^ Banha Teaching Hospital, Urology, Banha, Egypt

**Keywords:** fertility, smoking, semen

## Abstract

Our primary aim was to investigate the reversibility of the negative impact of smoking on male fertility. Secondarily, we studied the effect of both time after smoking cessation as well as the smoking index on semen quality improvement. Our study included 60 participants who applied to a smoking cessation program. The volunteers started by completing a fertility background questionnaire. Two semen specimens, provided 2 weeks apart, were analyzed according to the WHO guidelines for each candidate on three different occasions. The first was right before smoking cessation, followed by the 2nd and 3rd at 3 and 6 months after stopping smoking. Semen quality was compared between the three occasions. The degree of change was correlated with the level of smoking. The nicotine level in urine was used for smoking cessation compliance monitoring during the study. Comparing semen quality on the three different occasions, we detected a significant improvement in semen volume (3.33 mL ± 1.07 vs 3.88 mL ± 0.80 vs 4.34 mL ± 0.67, *P* < 0.001), sperm concentration (14.77 million /ml ± 6.49 vs 17.71 million/ml ± 6.41 vs 19.27 million/ml ± 6.46, *P* < 0.001), total sperm count (49.33 million ±25.31 vs 70.16 million ±30.85 vs 84.01 million ±31.00, *P* < 0.001), progressive motility (20.7 vs 35.3% vs 42.28%, *P* < 0.001), total motility (41.48 vs 60.33% vs 67.67%, *P* < 0.001), and abnormal morphology (69.32 vs 50.88% vs 40.82%, *P* < 0.001). Improvement in all parameters was positively correlated to time after cessation. Higher smoking indices were related to higher levels of improvement in semen volume, sperm concentration, and total count (areas under the curve of 0.867, 0.852, and 0.863, respectively, *P* < 0.001 for each). Our conclusion was that the negative effect of smoking on semen quality is reversible. Smoking cessation has a time-dependent positive effect on main semen parameters, hence positive potential for fecundity improvement in men.

**Lay summary:**

Extensive research efforts have always focused on the negative effects of smoking on male reproduction by comparing fertility in smokers versus non-smokers. There are very few studies looking at the effect of smoking cessation on male fertility. Our primary aim was to investigate whether the negative impact of smoking on male fertility was reversible. Our study included 60 participants who applied to a smoking cessation program. The volunteers started by completing a fertility background questionnaire. Each candidate provided two semen specimens 2 weeks apart, on three occasions. The first was right before smoking cessation, followed by the 2nd and 3rd at 3 and 6 months after stopping smoking. Semen quality was compared between the three occasions. The degree of change was correlated with smoking levels. We found the negative effects of smoking on semen are reversible.

## Introduction

Cigarette smoking remains one of the most prevalent lifestyle-related risk factors associated with a broad spectrum of human diseases (Kamimura *et al.* 2018). In recent years, increasing attention has been directed toward understanding its impact on male reproductive health (Kovac *et al.* 2015). Several studies, including meta-analyses (Sharma *et al.* 2016, Bundhun *et al.* 2019), have consistently demonstrated that smoking negatively affects semen quality, impairing parameters such as sperm concentration, motility, morphology, and volume (Mostafa *et al.* 2018, Osadchuk *et al.* 2023).

Furthermore, smoking has been linked to reduced sperm penetration capacity, decreased fertilization rates, and compromised DNA integrity, thereby raising significant concerns regarding its potential effects on fertility and offspring health (Price & Martinez 2020, Omolaoye *et al.* 2022). These deleterious effects are believed to stem from nicotine and a variety of toxic substances found in cigarettes, including heavy metals and reactive oxygen species (ROS) (Parameswari & Sridharan 2021, Bazid *et al.* 2022).

Despite the well-documented adverse impact of smoking on male fertility, there remains a significant gap in the literature regarding the potential reversibility of these effects following smoking cessation. Few studies have examined whether quitting smoking results in measurable improvements in semen parameters (Tang *et al.* 2019), highlighting a critical area in need of further research.

From a mechanistic perspective, cigarette smoke induces oxidative stress by increasing the production of ROS, to which spermatozoa are particularly vulnerable due to their high content of polyunsaturated fatty acids. Elevated ROS levels can lead to lipid peroxidation, particularly in the mitochondrial membrane, thereby impairing energy production, compromising membrane integrity, and reducing sperm mobility (Aboulmaouahib *et al.* 2018, Henriques *et al.* 2023). In addition, toxic metals such as cadmium and lead present in cigarette smoke are known to disrupt seminal zinc homeostasis and elevate oxidative stress, further impairing reproductive function (Machado-Neves 2022, Estevan *et al.* 2025).

Semen analysis remains the cornerstone diagnostic tool for evaluating male fertility, encompassing assessments of semen volume, viscosity, pH, sperm count, concentration, motility, morphology, and other qualitative parameters (Tanga *et al.* 2021, Esteves 2022). There is also growing evidence that smoking-induced DNA damage in sperm may contribute to gene mutations, chromosomal abnormalities, infertility, and an increased risk of miscarriage (Amor 2019). In light of these concerns, the present study aims to investigate whether smoking cessation leads to clinically meaningful improvements in semen parameters among previously active smokers.

## Patients and methods

**Figure 1:**
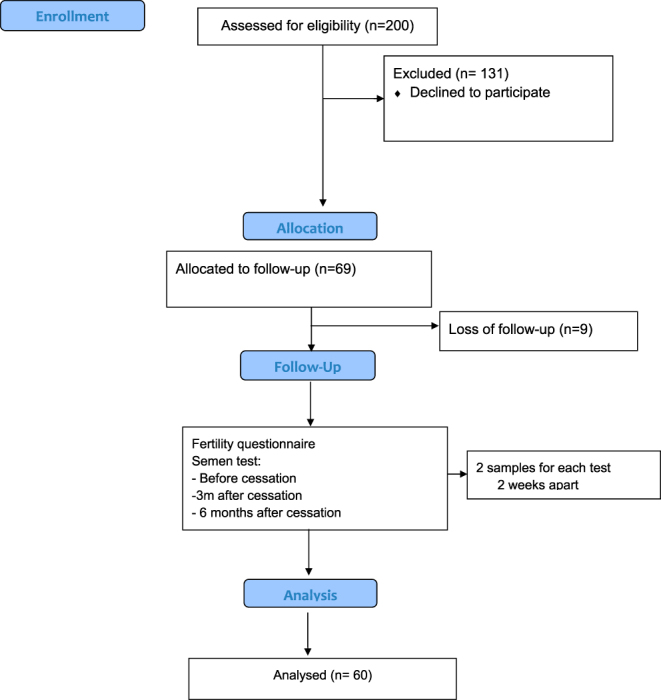
Flow diagram of the study.

This study was designed as a prospective cohort study with longitudinal follow-up. A total of 200 male smokers aged between 20 and 50 years, all seeking assistance with smoking cessation, were invited to participate. Of those, 69 individuals agreed to enroll in the study. During the follow-up period, nine participants dropped out, resulting in a final sample size of 60 participants who completed the study (Fig. 1).

The sample size calculation was performed using Minitab version 17.1.0.0 for Windows (Minitab Inc., 2013, USA). Based on a previous study by Vine *et al.* (1994) and focusing on semen volume as a key parameter of fertility, we assumed that a mean difference of 30 with a standard deviation of 60 would be clinically meaningful following smoking cessation. Using a significance level of 0.05 and a type II error of 20% (power = 80%), the minimum required sample size was determined to be 44 participants.

The study was conducted in the Urology Department, Faculty of Medicine, Beni-Suef University, in collaboration with the Special Chest Center in Mansoura. The research was carried out over a period extending from January 2021 to September 2022. Eligible participants were healthy male smokers between 20 and 50 years of age. Men who were found to be non-compliant with the smoking cessation protocol were excluded from the study.

Each participant began the study by completing a detailed fertility background questionnaire. Following this, they were asked to provide two semen samples, collected via masturbation, spaced 2 weeks apart, after a period of 3–7 days of sexual abstinence. Semen analysis was conducted according to the World Health Organization (WHO) 2010 criteria, assessing sperm count, motility, and morphology. Semen testing was repeated at 3 and 6 months following confirmed smoking cessation. Compliance with smoking cessation was verified by measuring urinary cotinine levels using a dipstick method, which provided a qualitative positive or negative result. Cotinine testing was conducted at baseline and repeated at 3 and 6 months to monitor adherence. In addition, detailed information regarding participants’ smoking behavior, including type, dose, and duration of smoking, was collected through a structured questionnaire and considered during the data analysis to control for potential confounding variables.

The study was initiated only after obtaining approval from the Research Ethics Committee of the Faculty of Medicine, Beni-Suef University (Approval No.: FMBSUREC/03112020/Mohammed). All participants were thoroughly informed about the objectives, methodology, and anticipated outcomes of the study. Written informed consent was obtained from all individuals before their enrollment.

### Statistical analysis

The statistical analysis was done using Minitab 17.1.0.0 for Windows (Minitab Inc., 2013, USA). Continuous data was presented as mean and SD or median and interquartile range (IQR), and categorical data as numbers and percentages (%). The normality of data was examined using the Shapiro–Wilk test. Independent *t*-test or Mann–Whitney test was used for comparison between two groups of continuous data, and chi-square test for comparison between two or more groups of categorical data. A paired t-test was used to compare two means before and after the intervention. All tests were two-sided, with *P* considered significant if < 0.05.

The smoking index was correlated with improvements in semen parameters. Smoking index = (number of packs per day) × (years of smoking). We assumed that ≥20% improvement of any parameter is considered significant using the Mann–Whitney U test. Our rationale relied on the fact that when a second semen analysis is done for confirmation of the first one and the difference is more than 20%, a third semen analysis is done for confirmation according to WHO rules for semen analysis. Hence, we inferred that more than 20% improvement could be considered a significant difference between two semen analyses. ROC analyses were done for the smoking index to predict improvements in semen parameters. Areas under the curve with 95% confidence intervals, best cutoff points, and diagnostic indices were calculated.

## Results

A prospective cohort study was conducted on 69 male participants who initiated a structured smoking cessation program, which included counseling and support groups. During the follow-up period, nine participants dropped out, resulting in a final sample of 60 individuals who completed the study. The mean age of participants was 32 ± 7 years, and their smoking index ranged from 2.5 to 60 pack-years. Over 70% were married, and 25% reported existing medical conditions such as hypertension, peptic ulcer disease, or chronic obstructive pulmonary disease (COPD). These baseline characteristics are presented in Table 1.

**Table 1:** Demographic characteristics of participants. Data are presented as mean ± SD or as *n* (%).

Characteristics	Values
*n*	60
Age, years	31.65 ± 7.35
BMI	26.99 ± 3.97
Smoking-index	16.39 ± 11.06
Marital status	
Married	42 (70)
Single	18 (30)
Medical problems	
HTN	5 (8.3)
Peptic ulcers	4 (6.6)
COPD	6 (10)

Baseline semen parameters, recorded before the initiation of the smoking cessation program, are detailed in Table 2. When comparing semen parameters over time, a statistically significant increase in semen volume was observed at 3 months post-cessation, with further improvement noted at 6 months (*P* < 0.001). In contrast, semen pH did not show a significant change throughout the study period (*P* = 0.26).

**Table 2:** Semen characteristic evaluation before starting the smoking cessation program.

Semen characteristics	Mean	SD	Minimum	Maximum
Volume	3.33	1.07	1.5	6
pH	7.50	0.17	7.25	7.9
Concentration (million/ml)	14.77	6.49	7	44
Total concentration	49.33	25.31	16	132
Progressive motility	20.70	6.47	8	39
Total motility	41.48	8.45	25	71
Total abnormalities	69.32	9.83	48	88
Head abnormalities	34.17	7.12	25	57
Mid piece abnormalities	12.62	2.81	5	20
Tail abnormalities	22.65	4.51	12	35
Vitality	42.03	8.37	25	70
Leukocytes	6.13	2.86	1	14
TZI	1.0085	0.129	1	1.02

Sperm concentration significantly increased at both the 3- and 6-month follow-up points (*P* = 0.001), as did the total sperm count (*P* < 0.001). Similarly, progressive sperm motility showed a significant and continuous increase over the same period (*P* < 0.001), as did total motility (*P* < 0.001). In terms of morphology, there was a significant decrease in total sperm abnormalities at both follow-up points (*P* < 0.001). Moreover, all morphological abnormality subtypes, head, mid-piece, and tail, exhibited consistent and statistically significant improvement after 3 and 6 months of smoking cessation (*P* < 0.001).

**Figure 2:**
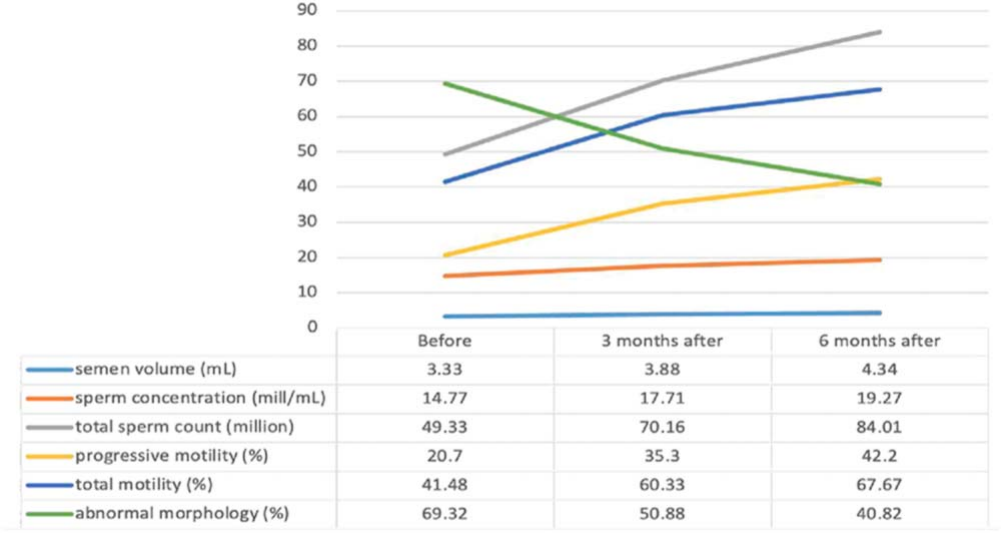
Semen quality change over time after smoking cessation.

Sperm vitality also improved significantly after 3 months of smoking cessation and continued to rise by the 6-month mark (*P* < 0.001). In addition, the semen leukocyte count showed a significant decrease after 3 months, with a further significant reduction observed at 6 months (*P* < 0.001), as illustrated in Fig. 2.

**Figure 3:**
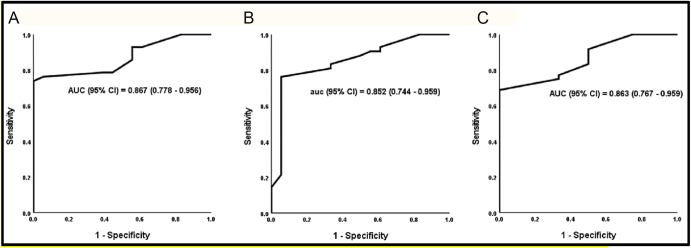
ROC analysis for the smoking index to predict improvement in semen parameters: (A) semen volume; (B) concentration; (C) total concentration.

Subgroup analysis revealed that the smoking index was significantly higher in participants who achieved a ≥20% improvement in semen volume compared to those with <20% improvement (median = 17.75 vs 10; *P* < 0.001). A similar pattern was observed among those with a ≥20% improvement in sperm concentration (median = 17 vs 9; *P* < 0.001) and ≥20% improvement in total sperm count (median = 15.5 vs 7.5; *P* < 0.001). However, no statistically significant differences in smoking index were detected in relation to changes in mid-piece abnormalities (*P* = 0.618), tail abnormalities (*P* = 0.383), or leukocyte count (*P* = 0.194), (Table 3). To evaluate the ability of the smoking index to predict improvements in semen parameters following smoking cessation, ROC curve analysis was performed (Fig. 3). The smoking index demonstrated strong predictive performance, with areas under the curve (AUC) of 0.867 for semen volume, 0.852 for sperm concentration, and 0.863 for total sperm count (*P* < 0.001 for all). These AUC values indicate high diagnostic accuracy, suggesting that individuals with a higher smoking index before cessation are more likely to experience significant improvements in semen quality.

**Table 3:** Smoking index according to semen parameter improvements.

Improvement in semen parameters	Median (min–max)	*P*-value
Improvement in volume		**<0.001**
≥20%	15.5 (5–60)	
<20%	10 (2.5–13)	
Improvement in concentration (million/mL)		**<0.001**
≥20%	17 (5–60)	
<20%	9 (2.5–25)	
Improvement in total concentration		**<0.001**
≥20%	15.5 (5–60)	
<20%	7.5 (2.5–12)	
Improvement in mid-piece abnormalities		0.618
≥20%	15 (2.5–50)	
<20%	13 (5–60)	
Improvement in tail abnormalities		0.383
≥20%	13.5 (2.5–50)	
<20%	21.25 (4–60)	
Improvement in leukocytes		0.194
≥20%	13 (2.5–60)	
<20%	18.75 (5–45)	

Bold *P* values indicate statistical significance.

The optimal cutoff point for predicting improvement in semen volume was identified as >13 pack-years, yielding a sensitivity of 73.8% and a specificity of 100%. For sperm concentration, the best threshold was >12 pack-years, with a sensitivity of 76.2% and specificity of 94.4%. Similarly, for total sperm count, a cutoff of >12 pack-years provided a sensitivity of 68.7% and specificity of 100%. These findings highlight the clinical value of the smoking index as a prognostic indicator, potentially aiding in counseling patients about expected fertility improvements following smoking cessation.

## Discussion

Smoking has long been associated with a range of adverse health outcomes, including detrimental effects on male reproductive health (Dai *et al.* 2022). The present study provides evidence that smoking cessation leads to significant improvements in semen quality, thereby contributing to the understanding of the reversible nature of smoking-induced reproductive damage. Given that spermatogenesis is a cyclical process occurring over approximately 74–90 days, these findings suggest that the negative impact of smoking on sperm parameters may be partially reversible within a few months of quitting.

A significant increase in semen volume was observed at both 3 and 6 months post-cessation. This improvement may reflect recovery of the accessory sex glands, or restoration of hormonal regulation affected by nicotine exposure (Laqqan *et al.* 2023). Previous research has similarly reported a negative correlation between semen volume and the number of cigarettes smoked daily (Asare-Anane *et al.* 2016).

Consistent with the existing literature, our study also demonstrated a significant increase in sperm concentration and total sperm count following smoking cessation. Nicotine and other toxicants in cigarette smoke are known to impair spermatogenesis, leading to lower sperm output (Holmboe *et al.* 2020). Furthermore, sperm motility showed marked improvement after quitting, aligning with earlier studies that attributed smoking-induced motility impairments to mitochondrial dysfunction caused by carbon monoxide and nicotine toxicity (Asare-Anane *et al.* 2016, Rahimi-Madiseh *et al.* 2020, Amor *et al.* 2022).

We also observed a significant reduction in sperm morphological abnormalities, particularly in the head, mid-piece, and tail regions. This may be attributed to decreased cotinine levels and the restoration of seminal zinc, which plays a crucial role in maintaining normal sperm structure. Although some studies have found no significant relationship between smoking and morphology (Gatimel *et al.* 2017), others have reported increased morphological abnormalities, especially in heavy smokers (Boeri *et al.* 2019).

In addition, sperm vitality improved significantly after 3 months of smoking cessation and continued to rise at 6 months. This improvement was paralleled by a significant reduction in seminal leukocyte count, which may reflect decreased oxidative stress due to a reduction in ROS. Elevated ROS levels are known to damage sperm membranes and DNA, particularly when antioxidant defenses are overwhelmed (Chianese & Pierantoni 2021).

Previous studies have associated higher smoking indices with greater semen deterioration (Sharma *et al.* 2016, Tang *et al.* 2019), our analysis applied ROC curves to determine whether the smoking index could predict a ≥20% improvement in semen quality. The optimal cut-off points identified were >13 pack-years for semen volume and >12 pack-years for sperm concentration and total concentration. These findings suggest that men with a higher smoking index may experience more pronounced improvements in semen parameters following cessation, possibly due to greater initial impairment and subsequent recovery. In this context, the values of 12 and 13 refer to smoking index thresholds (pack-years) derived from ROC analysis, representing the points of optimal sensitivity and specificity for predicting clinically meaningful changes in semen parameters.

Our findings align with those reported in previous research. For instance, a meta-analysis by Vine *et al.* (1994) documented a 17% reduction in sperm density among smokers, reinforcing the adverse impact of tobacco use on male fertility. Chia *et al.* (1994) also observed a correlation between smoking duration and reduced semen volume, along with increased abnormalities in the sperm head. Similarly, Zhang *et al.* (2000) reported dose-dependent declines in sperm density, viability, morphology, and motility among smokers. In a study by Künzle *et al.* (2003), smokers exhibited over 15% lower sperm density, 16% lower motility, and 10% fewer morphologically normal sperm compared to non-smokers. More recently, Chua *et al.* (2023) and Kulaksiz *et al.* (2022) reaffirmed these patterns, further supporting the detrimental effects of smoking on sperm quality.

Additional studies have identified smoking-related sperm abnormalities, such as head defects and cytoplasmic droplets (Zenoaga-Barbăroşie & Martinez 2024). A pooled analysis by Amoah *et al.* (2025) indicated that heavy smokers had 19% lower sperm density and exhibited decreased testosterone levels. Surach (2022) further distinguished that motility was more affected in light smokers, whereas morphology deteriorated more significantly in heavy smokers. Ranganathan *et al.* (2019) also emphasized the impact of smoking on sperm motility and DNA integrity. However, contradictory results have emerged in the literature. For example, Saleh *et al.* (2002) found no significant differences in semen parameters or leukocyte counts between smokers and non-smokers, and Dikshit *et al.* (1987) reported only minimal declines among smokers and tobacco chewers. Some have suggested that smoking may not substantially influence semen quality in men with idiopathic infertility (Singh 2014).

In the context of assisted reproductive technology (ART), the implications of smoking are even more pronounced. Long-term smoking in women (more than 5 years) has been associated with reduced oocyte yield and lower implantation rates, while male smoking has been shown to affect sperm count, motility, and DNA integrity (Ma *et al.* 2023). Notably, Vanegas *et al.* (2017) reported that ART outcomes improved in men who quit smoking, demonstrating a 4% reduction in ART failure risk for each year of smoking cessation.

To our knowledge, this is a unique study, as it is the first study to explore the relationship between smoking index and the magnitude of semen parameter improvement after smoking cessation. While most previous studies have compared smokers with non-smokers, relatively few have explored outcomes following smoking cessation. One such study by Kulaksiz *et al.* (2022) followed infertile men for 3 months and observed improvements in semen volume and count. Our study is the first to build upon this by including both fertile and infertile men, extending the follow-up period to 6 months, and using biochemical verification (urinary cotinine) rather than relying solely on self-reported smoking status. In addition, we found significant improvements not only in volume and count, but also in sperm motility and morphology, making our findings more comprehensive and robust.

Future studies should consider long-term reproductive outcomes, including pregnancy and live birth rates, as primary endpoints. In addition, incorporating hormonal profiles and objective nicotine biomarkers would strengthen mechanistic understanding.

### Limitations

A significant limitation of our study is the absence of fertility outcomes, such as pregnancy or live birth. Improvements in semen parameters may not guarantee conception. Future research should incorporate live birth rates to better assess the reproductive benefits of smoking cessation.

Other limitations include the absence of a non-smoking control group and potential confounding factors, such as diet or occupational exposure. Furthermore, hormonal assessments (e.g. testosterone, LH, FSH) were not conducted and may offer valuable mechanistic insights. Despite these limitations, our study underscores the clinical relevance of smoking cessation and its potential role in improving male reproductive health.

## Declaration of interest

The authors declare that there is no conflict of interest that could be perceived as prejudicing the impartiality of the research reported.

## Funding

This research did not receive any specific grant from any funding agency in the public, commercial, or non-profit sectors.

## Author contribution statement

MM (corresponding author) was responsible for conceptualization, study design, supervision, data interpretation, and manuscript drafting. AR helped in data collection, statistical analysis, manuscript writing, and critical revision. AA was responsible for patient recruitment, data acquisition, and contribution to manuscript editing. AM contributed to clinical evaluation, coordination of follow-up, and manuscript review. AE was responsible for data analysis support, literature review, and final approval of the manuscript.
